# Anti-Inflammatory and Immunoregulatory Action of Sesquiterpene Lactones

**DOI:** 10.3390/molecules27031142

**Published:** 2022-02-08

**Authors:** Ana Paço, Teresa Brás, Jacqueline O. Santos, Paula Sampaio, Andreia C. Gomes, Maria F. Duarte

**Affiliations:** 1 Alentejo Biotechnology Center for Agriculture and Agro-Food (CEBAL)/Polythechnic Institute of Beja (IPBeja), 7801-908 Beja, Portugal; anaisapaco@gmail.com (A.P.); teresa.bras@cebal.pt (T.B.); jacqueline.oliveira@cebal.pt (J.O.S.); 2 Mediterranean Institute for Agriculture, Environment and Development—MED, CEBAL, 7081-908 Beja, Portugal; 3 Centre of Molecular and Environmental Biology (CBMA), Department of Biology, University of Minho, Campus of Gualtar, 4710-057 Braga, Portugal; psampaio@bio.uminho.pt (P.S.); agomes@bio.uminho.pt (A.C.G.)

**Keywords:** anti-inflammatory action, immune response, JAK-STAT, MAPK, NF-κB, sesquiterpene lactones, structure–activity relationship

## Abstract

Sesquiterpene lactones (SL), characterized by their high prevalence in the *Asteraceae* family, are one of the major groups of secondary metabolites found in plants. Researchers from distinct research fields, including pharmacology, medicine, and agriculture, are interested in their biological potential. With new SL discovered in the last years, new biological activities have been tested, different action mechanisms (synergistic and/or antagonistic effects), as well as molecular structure–activity relationships described. The review identifies the main sesquiterpene lactones with interconnections between immune responses and anti-inflammatory actions, within different cellular models as well in in vivo studies. Bioaccessibility and bioavailability, as well as molecular structure–activity relationships are addressed. Additionally, plant metabolic engineering, and the impact of sesquiterpene lactone extraction methodologies are presented, with the perspective of biological activity enhancement. Sesquiterpene lactones derivatives are also addressed. This review summarizes the current knowledge regarding the therapeutic potential of sesquiterpene lactones within immune and inflammatory activities, highlighting trends and opportunities for their pharmaceutical/clinical use.

## 1. Introduction

Inflammation could generally be defined as a protective response by an organism triggered by pathogens or endogenous stress signals [1]. Immune cells, mostly myeloid cells, can specifically recognize pathogen-associated molecular pattern molecules (PAMPs) or damage-associated molecular patterns (DAMPs), and initiate mechanisms to eliminate the initial trigger, after which, the inflammatory process must be adequately resolved. When allowed to continue unchecked, inflammation may result in autoimmune or autoinflammatory disorders, neurodegenerative diseases, or even cancer.

The immune system is a complex network of protein and cells interactions in differentiated organs and tissues, with the goal to protect the organism from diseases and substances identified as “non-self”. This system comprises a diversity of different cell types and proteins. Each performs a specific mission, collaborating in a magnificent way to the recognition and reaction against “non-self” [2]. Despite all elements of the immune system interacting with each other, two types of immune responses can be considered: the innate immune response and the acquired immune response [3]. Innate immune responses are carried out by cells that do not need previous activation to reach their maximum response. These cells include neutrophils, monocytes/macrophages [4], eosinophils, and basophils [5,6]. Neutrophils are the main mediators of a rapid innate host defense against most bacterial and fungal pathogens, while natural killer (NK) cells are important in the early stages against intracellular pathogens, particularly killing virally infected cells [7]. These immune cells have granules and are the most abundant of all white blood cells in humans, killing microorganisms by microbicidal agents present in its granules and others produced during activation [8]. Monocytes/macrophages are white blood cells of the immune system, present in the bloodstream and tissues, respectively, which engulf and digest microbes, cellular debris, and other foreign substances that affect the health of a high number of organisms. This process, common to neutrophils, is named phagocytosis, and acts to defend the host against infection and injury [9]. Most macrophages are located at strategic points in the host organism where microbial invasion or accumulation of foreign substances are likely to occur [10]. The basophils and eosinophils correspond to a low percentage of white blood cells, having a role more specific in allergic reactions and parasitic infections [5,6]. In fact, it was suggested that eosinophils and basophils may mainly act as regulatory cells in immune responses, in the context of allergy or parasitic infections, rather than effector white blood cells, such as neutrophiles and monocytes/macrophytes [11,12].

Unlike innate immune responses, the adaptive responses are highly specific to the particular pathogens/antigens that induce them. The acquired immune system is already present at birth; immune memory formation only occurs after exposure of adaptive cells to the specific antigen. This exposure occurs throughout life, but the first three years of life is a critical period where most naive lymphocytes (B and T lymphocytes) are activated, greatly enhancing immune memory formation [13]. In autoimmune diseases, such as rheumatoid arthritis and multiple sclerosis, due to the persistence of autoantigen, autoreactive T and B cells will be activated and maintained.

Mechanisms involving the entire inflammatory process, as well an individual’s immunomodulation response capacity, are not completely understood, but highlight extremely important cellular players, as well as multiple regulatory levels. Currently, there are a considerable number of anti-inflammatory medicines, with different therapeutic strategies that may be summarized as: (i) reducing the activity of specific cytokines or their receptors (e.g., P2 × 7 receptor inhibitors in certain viral infections [14]; cytokine suppressive anti-inflammatory drugs (CSAIDs) inhibiting NF-κB and p38 MAPK signaling to avoid pre-term birth); (ii) blocking lymphocyte trafficking into tissues (e.g., vedolizumab, an anti-α4β7 integrin, for treating Crohn’s disease [15]); (iii) prevent the binding of monocyte-lymphocyte costimulatory molecules; and (iv) deplete B lymphocytes (monoclonal antibodies against CD20**,** rituximab, for non-Hodgkin lymphoma [16,17]). Prolonged exposure to anti-inflammatory drugs, such as glucocorticoids and nonsteroidal anti-inflammatory drugs, has been described as having considerable side effects, such as susceptibility to secondary infections. The pharmaceutical companies still search for the development of more effective and less toxic anti-inflammatory agents, addressing other molecular responses, to treat either acute inflammation or chronic inflammatory diseases. 

Nature is a rich source for compounds with anti-inflammatory properties. This recognition is nowadays underlined by the significant number (~25%) of FDA-approved natural anti-inflammatory drugs, which are natural product derivatives [18]. From all secondary metabolites that can be found in plants, the sesquiterpene lactones (SL) group is one of the most prevalent and biologically significant, comprising over 5000 known compounds [19]. With new SL discovered in the last years, new biological activities have been tested, as well as different mechanisms of action (synergistic and/or antagonistic) and structure–activity relationships. SL exhibited a wide range of biological activities with impacts in human health, ranging from antitumor, antimicrobial, antioxidant, hepatoprotective, among many others, reported within a large list of published manuscripts. This review summarizes the current knowledge regarding SL therapeutic potential regarding inflammation activities, as well as highlights the responses that can be induced towards the immune system, aiming to bring into discussion the trends and opportunities for SL pharmaceutical/clinical use, and awareness about putative harm effects. 

The methodology applied in the present review was an examination of literature conducted in 2021 between March and November via electronic searches, using Scopus, Web of Science, Science Direct, Google Scholar, ClinicalTrials.gov, www.drugbank.ca, and https://newdrugapprovals.org/, (accessed on 8 December 2021), and publications in peer-reviewed scientific journals. The keywords used were structure–activity relationship, inflammation, immune system, Nf-kB, MAPK, and JAK-STAT signaling mechanisms, all combined with sesquiterpene lactones and/or sesquiterpene lactones derivatives. The scientific names were validated by using Plant List, available online: www.theplantlist.org (last accessed on 9 January 2022), International Plant Name Index, and Kew Botanical Garden databases. The literature mentioned in this paper dated from 1971 to 2021 and were limited to the English language. The final data collected through the authors’ discussions were then compiled, evaluated, compared, and conclusion were drawn accordingly.

## 2. Sesquiterpene Lactones and Their Structure–Activity Relationships

Sesquiterpene lactones (SL) are a major group of secondary metabolites found in plants [19] and could generally be included in the *Cactaceae*, *Solanaceae*, *Araceae,* and *Euphorbiaceae* families, with a high prevalence in *Asteraceae*, where they can be found all over [19].

The medicinal properties of SL have been used since immemorial times, initially without the specific knowledge of what were SL. In folk medicine, it was usually used as part of the plants to treat numerous diseases [19]; for example, using boiled leaves of the plant *Artemisia douglasiana* in the treatment of gastric ulcers. These leaves present dehydroleucodine—SL with proven effects in the treatment of peptic gastric ulcers [20]. SL biological activities are associated with adjuvant treatments for a wide range of diseases, such as cancer and cardiovascular diseases, and neurodegenerative diseases, such as Alzheimer’s and Parkinson’s [21,22], as well as malaria, diarrheal, viral infections (influenza, herpes simplex virus, SARS-CoV-2)) [23], bacterial infections, migraines, and rheumatoid arthritis. They are even used to treat insect bites, presenting analgesic and sedative effects [24,25,26]. 

**Figure 1:**
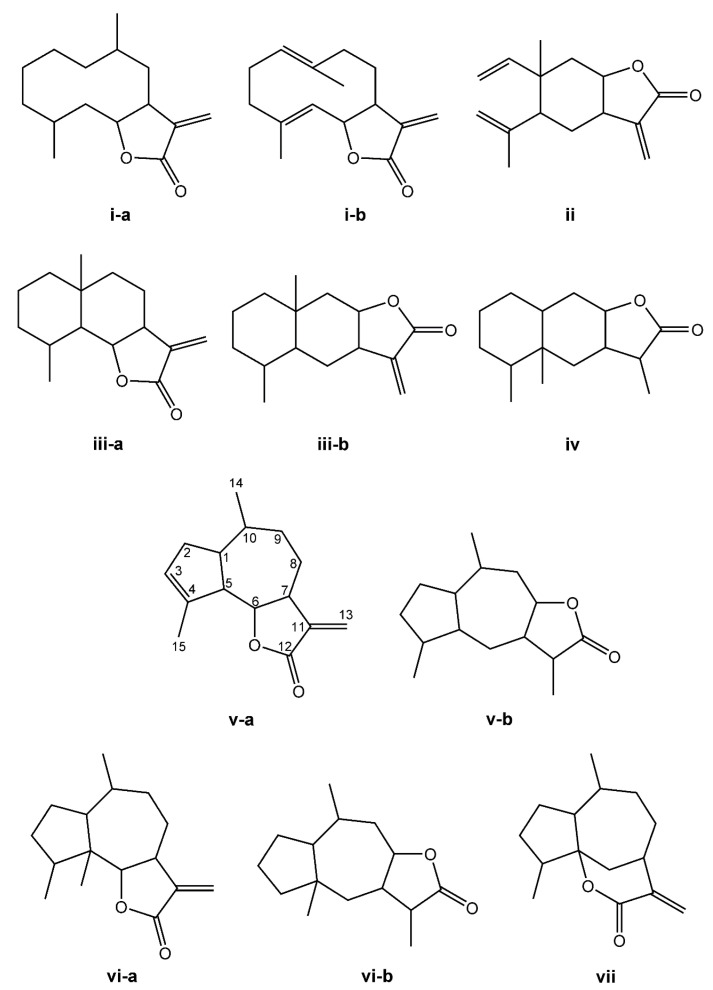
Structures of the main sesquiterpene lactone-type skeletons: germacranolide isomers (**i-a**,**i-b**); elemanolide (**ii**); eudesmanolide isomers (**iii-a**,**iii-b**); eremophilanolide (**iv**); guaianolide isomers (**v-a**,**v-b**); pseudoguaianolide isomers (**vi-a**,**vi-b**); and hypocretenolide (**vii**).

The SL are derived from two main precursors—isopentenyl diphosphate (IPP) and dimethylallyl diphosphate (DMAPP) [27]. These precursors can be generated in plants via either the mevalonate pathway (MVA), which occurs within the cytosol, or the 2-C-methyl-D-erythritol (MEP) pathway, occurring in the chloroplasts [28,29]. IPP and DMAPP are converted into farnesyl diphosphate (FPP) by the enzyme farnesyl diphosphate synthase [27]. FPP is considered a common precursor for SL, but can be further converted into sterols, triterpenes, or used for prenylation of proteins. This terpene subclass can be organized based on a carbon-cyclic skeleton (Figure 1), as follows: (i) germacranolides (with a ten-membered ring); (ii) elemanolides (with a six-membered ring); (iii) eudesmanolides and (iv) eremophilanolides (both with six-membered rings); and (v) guaianolides; (vi) pseudoguaianolides; (vii) hypocretenolides (with five- and seven-membered rings, with a methyl group at the C-4 or C-5 position) [30,31].

**Figure 2:**
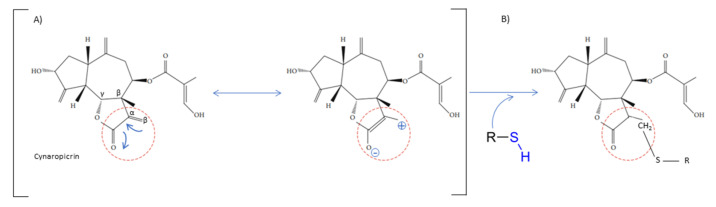
Cynaropicrin chemical structure, with the reactive center, α-methylene-γ-lactone moiety (αM γL). The reactive centers of SL are evidentiated with red circles. (**A**) Michael reaction between α-methylene-γ-lactone moiety (αM γL). (**B**) Reaction with a sulfhydryl group.

The chemical structure shares an oxygen-containing ring structure with a carbonyl function and structure–activity relationship (SAR) profile studies, attributed to the α-methylene-γ-lactone group (αM γL) and a wide range of SL biological effects, as it exerts activity by means of alkylation of thiol groups, commonly found in proteins (Figure 2) [32], demonstrated to be crucial for SL inhibitory effects upon different molecular processes. After 1991, interest in SL structure–activity relationships regained with the advent of novel methods, namely comparative molecular field analyses, quantitative fractional accessible molecular surface areas, self-organizing maps, and molecular descriptors, clearly associated to an increase in publications related to SL structure–activity relationships. According to Choodej et al., costunolide and eudesmanolide-type sesquiterpene derivatives, when synthesized with no αM γL moiety in their structures, do not show any detectable activity in terms of decreasing TNF-α, even at high concentrations (50 µmol/L) [33], in comparation to original costunolide with an IC_50_ value of 2.05 µmol/L [33], showing the essential role of αM γL moiety for the anti-inflammatory effect on TNF-α secretion in activated macrophages. Cynaropicrin also known to inhibit TNF-α and NO production in a dose-dependent manner [34], when treated with SH compounds (i.e., L-cysteine, dithiothreitol, and 2-mercaptoethanol) loses the inhibitory effect upon TNF-α and NO, suggesting that cynaropicrin anti-inflammatory activity is mediated by conjugation with SH-groups of target proteins [35] (Figure 2). More than 300 mM of cysteine (15-fold as a molar ratio between cynaropicrin and L-cysteine) attenuates the suppressive effect of cynaropicrin up to 90%, not only suggesting that a high molar ratio is required to completely abrogate the cynaropicrin effect, but also that the binding affinity between cynaropicrin and the target protein might be higher than that between cynaropicrin and L-cysteine [34].

SAR profile studies have also demonstrated that SL αM γL moiety combined with a C_4_-C_5_ epoxide ring can interact with the sulfhydryl groups through the αM γL [36], as well as with the hydroxyl and amine groups through the epoxide ring. The use of a prodrug approach, by addition of an amine group into the αM γL moiety, leads to amino-derivatives with similar biological activities, increased solubility, and improves the selectivity by reducing unspecific binding to biological thiols via the Michael-type addition to the αM γL moiety. Similar approaches were successfully applied to several SL, namely helenalin, costunolide, and parthenolide [37]. The SAR studies also demonstrated that an ester group at C-8 might be more important than the αM γL moiety for SL cytotoxicity, as it was demonstrated with 11,13-β-dihydro-lactucopicrin, lacking the αM γL moiety, but carrying an ester group at C-8, was more cytotoxic to nasopharyngeal and liver cancer cells than lactucin (the same structure but with an αM γL moiety and no ester at the C-8 position) [38]. 

SL carbon-cyclic skeleton molecular geometry organizations also imprint different biological activities as the structural and chemical natures also change. The germacranolides, with ten-membered rings, more easily adapt to different conformation structures and therefore turn out to be more available to interact with different biological targets, comparatively to eudesmanolides (with six-membered rings), which are more restricted in their bioactivities. The example of heliangolides, containing furane rings, have been described as more effective than guaianolides, on account to their greater conformational flexibility. Studies of structurally-related pseudoguaianolides showed that the β-OH isomer (parthenin) at C-1 is more active against ethyl phenylpropiolate-induced mouse ear edema than the α-OH equivalent (hymenin) [39]. 

The different studies developed over the last years using SL-derivative strategies, demonstrated the importance of different SL reactive centers, their impacts upon interaction with biological targets, as well as their capacity to potentiate their clinical relevance, namely increasing aqueous solubility, diminishing toxicity, acquiring better pharmacokinetics, among many other important features [40].

## 3. Sesquiterpene Lactones in Medicine: Immunoregulatory Response and Anti-Inflammatory Activities

In 2010, the SL in clinical trials were artemisinin, thapsigargin, and parthenolide [41]. Ten years later, in 2020, some SL isolated from the *Asteraceae* species, were already commercially available, such as artemisinin and parthenolide [41,42]. Moreover, the SL alantolactone, arglabin, costunolide, cynaropicrin, helenalin, inuviscolide, lactucin, parthenolide, thapsigargin, and tomentosin are use in in vivo studies, preclinical, and few in clinical studies. These SL show promising anti-inflammatory effects and their immunoregulatory effects deserve attention, aside from their many biological activities [43]. The cellular and molecular activities of SL will be described in detail below.

### 3.1. Immunomodulatory Effects of Sesquiterpene Lactones at the Cellular Level

Stimulation of immune cells lead to active inflammation through cytokine-mediated actions. Table 1 summarizes the direct activities that have been described so far, of different SL on immune cells (and key cytokines) involved in innate and acquired immune responses. Artemether, an artemisinin derivative, was found to significantly suppress the proliferation, IL-2, and interferon-γ (IFN- γ) production by T cells triggered by T cell receptor engagement [44]. Artemether significantly inhibited the T cell receptor engagement-triggered MAPK signaling pathway, including phosphorylation of ERK1/2, JNK, and p38. The authors further dissected that artemether majorly affecting the function of T cells, rather than the antigen presenting cells to exert the immunosuppressive effects [44]. Cynaropicrin presented equivalent effects upon T cell proliferation from splenocytes, as demonstrated by Cho et al. The authors studied cynaropicrin anti-mitogenic effects upon T- and B lymphocytes treated with concanavalin A, phytohemagglutinin, and lipopolysaccharide. In all cases, there was a decrease in T cell proliferation (either CD4^+^ or CD8^+^), known to play a crucial role in chronic inflammatory processes though activation of inflammatory mast cells, eosinophils, neutrophils, and macrophages, resulting in a massive production of chemical mediators and pro-inflammatory cytokines. Schepetkin et al. (2018) tested thirteen SL in regard to reduction of T cell activation [45]. The authors concluded that five SL, the arglabin, grosheimin, agracin, parthenolide, and estafiatin, could inhibit T lymphocytes receptors, therefore having immunotherapeutic properties (Table 1). Recently, the effects of 7-hydroxyfrullanoide in inhibiting CD4^+^ T cells and peritoneal macrophage responses were investigated. The 7-hydroxyfrullanoide reduced IL-2 and simultaneously induced Ca^2+^ (an intracellular chelator, which lowers lactate and rescues CD4^+^ T cell cycling) [46]. Moreover, intraperitoneal administration of 7-hydroxyfrullanoide lowers serum inflammatory cytokines IFN- γ, IL-6, reduces the effects of dextran sulfate sodium-induced colitis, and emphasizes the anti-inflammatory potential of 7-hydroxyfrullanoide in lowering immune responses [46]. 

Abe et al. (2015) reports a role of the SL tagitinins isolated of *Tithonia diversifolia* in activation and survival of human neutrophils [47]. Tagitinins C, F, and A decrease IL-6, IL-8 and TNF-α production by human neutrophils (Table 1), but only tagitinin F did so safely, without inducing neutrophil apoptosis. Aerial parts of *Inula hupehensis Ling*. have a diversity of SL (eudesmanolides, germacranolides, and xanthanolide), all with an inhibitory effect against LPS-induced nitric oxide (NO) production in macrophages [48,49] (Table 1). Moreover, Lee et al. (2018) reports in rats that the alantolactone, costunolide, and dehydrocostus lactone isolated from *Saussurea costus (Falc.) Lipsch* have roles in allergic asthma, by reducing the number of immune cells, particularly eosinophils, underlying SL role in allergic immunity [50] (Table 1). Moreover, in rats, the inhibitory effects of SL isolated from *Eupatorium chinense* L. on IgE-mediated degranulation of basophils, was reported [51]. These immune cells keep their specific secretory immune products in granules and may release them by exocytosis during an inflammatory response [52]. Therefore, the inhibition of their degranulation reduces the immune response of these cells. The effectiveness of some rich extracts of SL, e.g., *Vernonia scorpioides* L. ethanolic extracts, containing diacethylpiptocarphol and hirsutinolides, were tested when applied topically in acute and chronic cutaneous inflammation models in mice. The results demonstrated that topical application of ethanolic extract of *Vernonia scorpioides* L. reduced edema and induced myeloperoxidase activity to a comparable value to the reference drug dexamethasone, a corticosteroid [53], meaning a reduction of neutrophil infiltration. Recently, an in vivo study using lychnopholide, eremantholide C, and goyazensolide, three sesquiterpene lactones extracted from *Lychnophora* species, were assessed regarding their anti-inflammatory actions, using a monosodium urate (MSU) crystal-induced arthritis C57BL/6 mice animal model. The tested SL exerted anti-inflammatory effects by inhibiting neutrophil migration and blocking the release of TNF-α [54]. Budlein A, a SL from *Viguiera robusta*, presented an in vivo response in a model of acute gout arthritis in mice. Budlein A reduced neutrophil recruitment, phagocytosis of MSU crystals by neutrophils, and Il-1β and TNF-α mRNA expression in the knee joints. In vitro, budlein A decreased TNF-α production, which might be related to the inhibition of NF-κB activation. Furthermore, budlein A reduced the IL-1β maturation, possibly by targeting inflammasome assembly in macrophages [55]. Similar results were recently obtained with alantolactone in a collagen-induced arthritis DBA/1 mouse model. Alantolactone at 50 mg/kg attenuates rheumatoid arthritis symptoms, including high arthritis scores, infiltrating inflammatory cells, synovial hyperplasia, bone erosion, and levels of the proinflammatory cytokines TNF-α, IL-6, and IL-17A, but not IL-10 in paw tissues. The number of splenic Th17 cells and the capability of native CD4^+^ T cells to differentiate into the Th17 subset (one of the rheumatoid arthritis pathogenic pathways), by downregulating STAT3/RORγt signaling by as early as 24 h of treatment, was also achieved by alantolactone treatment [56]. Alantolactone therapeutic effects underlie the suppression of inflammatory cytokines and the modulation of immune response. 

### 3.2. Overview of Main Signaling Pathways Involved in Inflammatory Responses Modulated by Sesquiterpene Lactones

The inflammatory immune response (IIR) is a physiological or systemic reaction against harmful stimuli, as pathogens, damaged cells, and toxins [60], mainly played by immune cells, such as monocytes/macrophages, lymphocytes, neutrophils, and dendritic cells [61,62]. The purpose of IIR is to eliminate the initial cause of cell injury. The inflammatory response can be classified as acute and chronic, the first contributes to repair of tissue homeostasis, culminating in the resolution of the acute inflammation. Nevertheless, uncontrolled acute inflammation could become chronic, impacting a variety of chronic inflammatory diseases [63]. Several IIR pathways were described up to now, namely the NF-κB pathway, MAPK pathway, and JAK-STAT pathway [64], which can be activated in cells with contact to external agents, as the epidermal keratinocytes of skin [65], lung, and intestinal epithelial cells [66,67], as well as in leukocytes from blood. These signal pathways in non-immune cells have several effects in immune cells (such as macrophages), which impact the inflammatory process. Due to the complexity of the inflammatory process, below, we only present some examples of how these signal pathways affect immune cells. As the focus of this review is to explore the role of the SL in the immune response and anti-inflammatory activity, here we present a brief explanation of these pathways and how SL can interact and regulate them.

**Figure 3:**
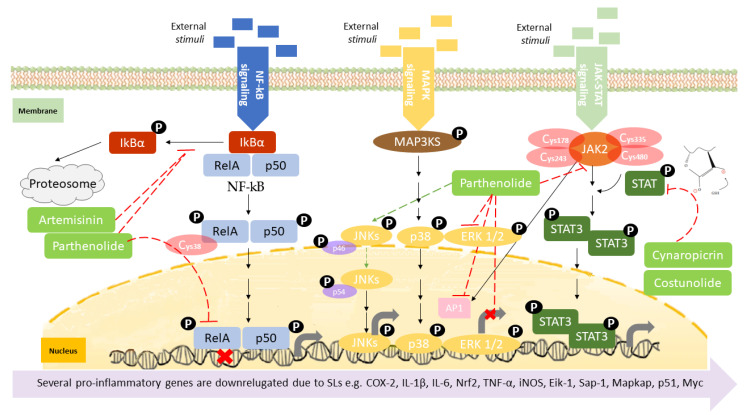
Overview of the three major signaling pathways regulating inflammation: NF-kB, MAPK, and JAK-STAT. Few sesquiterpene lactones are presented as examples to demonstrate their inhibitory action within the different molecular mechanisms.

Canonical nuclear factor κB (NF-κB) signaling relies on transcription factors, including p50 and RelA (p65) [68], which mediates the immune response by controlling gene expression that favors inflammatory response [61,64]. The activation of the canonical NF-kB is stimulated by the reception of external stimuli in the cell surface, resulting in a double phosphorylation of the inhibitor of nuclear factor kappa B (IkBα) in the cytoplasm by the I-kappa-kinase (IKK), which results in its degradation, releasing RelA and P50 (Figure 3). These two proteins enter in the nucleus controlling gene expression [61], namely several pro-inflammatory genes, including those coding for cyclooxygenase-2 (COX-2), tumor necrosis factor-alpha (TNF-α), inducible nitric oxide synthase (iNOS), and interleukin 1 beta (Il1β), among many others [69], affecting immune response, cell apoptosis, cell-cycle progression, inflammation, and oncogenesis [70]. NF-kB activation is also associated with activity and chemoattraction of macrophages, chemoattraction of T lymphocytes, survival of neutrophil, and maturation of dendritic cells [71,72,73,74]. The action mechanism of certain SL upon NF-kB has been extensively studied. Parthenolide is recognized as a potent anti-inflammatory agent [75], its bioactivity being driven by the capacity to deregulate the NF-kB signaling pathway, in concentrations as low as 5 µM [41]. As described by Garcia-Pineres et al., the high binding selectivity allows parthenolide to form a covalent bond with cysteine-38 of the NF-kB RelA subunit [76], leading to its alkylation and, consequently, inhibition of NF-kB DNA binding (Figure 3) (Table 2). The same germacrolide was also described as potentiating the accumulation of IkBα in cystic fibrosis cells, with simultaneous inhibition of NF-kB translocation, associated to a significant decrease in IL-8 expression [77] (Figure 3). Artemisinin, a germacrolide, was able to inhibit the LPS/cytokine-induced increase of NOS activity, promoted by inhibition of NF-kB nuclear translocation. As described by parthenolide, artemisinin also shares the ability to prevent the LPS-induced proteolytic degradation of IkBα protein and the consequent activation and translocation of NF-kB, accounting for the suppression of iNOS gene expression and NO synthesis in cells stimulated with LPS/cytokines [78] (Figure 3) (Table 2). Braquet et al. (1985) reported that the SL of *Ginkgo biloba*, ginkgolides, favor the prevention of platelet aggregation and thrombosis [79] (Table 2). The platelet-activating factor (PAF) signaling pathway plays a key role in the initiation and progression of inflammatory and thrombotic reactions, by NF-kB and MAPK signal pathways [80], with the ginkgolides having the ability to antagonize PAF-induced platelet aggregation [81]. (Table 2). Santamarin, a sesquiterpene lactone isolated from *Aucklandia lappa* (*Asteraceae*), increases heme oxygenase-1 expression by Nrf2 translocation and suppression of NO, PGE2, TNF-α, and IL-1β production through NF-kB inhibition in LPS-induced macrophages (Table 2) [82]. Recently, the suppressive effect of dehydrocostus lactone, upon signal transduction via toll-like receptor (TLR) signaling pathway, demonstrated this sesquiterpene lactone as an effective downregulator of NF-kB and interferon regulatory factor 3, the representative transcriptions factors involved in the inflammatory response, induced by TLR agonists, as well as diminished expression of COX-2 and interferon inducible protein-10 [83] (Table 2). Weng et al. described, very recently, the inhibitory action of lactucopicrin, a bitter sesquiterpene lactone of leafy vegetables, such as chicory, curly escarole, and lettuce, upon vascular endothelial NF-kB. The inhibitory effect of lactucopicrin was not due to modulation of IKK-, IkBα-, or NF-kB RelA-binding activity. Lactucopicrin reduced importin-α3 mRNA stability by 60.6%, resulting in reduced steady-state expression of importin-α3 in inflammatory human aortic endothelial cells (Table 2). Importin-α3 escorts Nf-kB to translocate to the nucleus [84], where it drives the expression of a wide range of genes, including the vascular cell adhesion molecule 1 (VCAM-1) and intercellular adhesion molecule 1 (ICAM-1), known to be elevated in patients with either acute or chronic vascular inflammatory diseases. Thus, lactucopicrin reduces vascular endothelial inflammation through inhibiting NF-kB activation promoted by importin-α3 downregulation [84]. 

The MAPK signaling pathway is initiated by the reception of external stimuli, as mitogens, which allow the phosphorylation and activation of a protein cascade. This pathway comprises at least three components, a mitogen-activated protein kinase (MAPK), a MAPK kinase (MAPKK), and a MAPK kinase kinase (MAPKKK). MAPKKKs phosphorylate and activate MAPKKs, which in turn phosphorylate and activate MAPKs [85]. Activation of the MAPK leads to phosphorylation and activation of ERK, JNK, and p38 transcription factors, which initiate the inflammatory response [86], controlling the expression of a high variety of genes, such as ElK-1, Sap-1, and MAPKAP [87], resulting in the control of cell proliferation, apoptosis, and differentiation (Figure 3) [88]. Sesquiterpene lactone also has role in the inhibition of the MAPK signaling pathway activation. Parthenolide, besides the effect upon NF-κB described above, also inhibits the activation of the MAPK pathways, specifically constraining the activation of the ERK [89] (Table 2). Since MAPKs ERK and JNK activate AP-1, Saadane A et al. demonstrated that parthenolide inhibited phosphorylation and activation of ERK1 and ERK2 (Figure 3). Parthenolide did not alter the expression of all downstream MAPK players, as it was described that parthenolide stabilized p46 JNK phosphorylation as well as p38 in cystic fibrosis cells. Moreover, achillolide A, a sesquiterpene lactone isolated from *Achillea fragrantissima*, was assayed in terms of the phosphorylation effect upon SAPK/JNK and p44/p42 MAPK using Neuro2a cell model. The results highlight achillolide A efficacy, inhibiting phosphorylation of SAPK/JNK and p44/p42 MAPK and, consequently, downregulating MAPKs [90] (Table 2). Alantolactone, isolated from the root of *Aucklandia lappa*, in LPS-stimulated RAW264.7 cells and peritoneal macrophages, inhibited iNOS, COX-2, as well as the downstream products, nitric oxide, prostaglandin E_2_, and tumor necrosis factor-α. These effects were promoted by the inhibitory activity of alantolactone upon NF-kB signaling and inhibition of IkBα phosphorylation and IKK. At the same time, it also inhibits MAPK phosphorylation, including JNK, ERK, and p38, promoted by the MyD88 signaling pathway [91] (Table 2).

In the JAK-STAT pathway, the Janus kinases (JAKs) are activated by ligands on the cell surface receptors, as growth factors; then JAKs add phosphates to the receptor. This allows the recruiting of two cytoplasmic signal transducers and activators of transcription factor proteins (STATs) that bind to the phosphates, forming a dimer. The STAT dimer translocate into the nucleus-binding target gene promoter regions, to regulate transcription of inflammatory genes [92,93] (Figure 3), such as cytokines, (as IL-6) and growth factors, as well as protooncogenes (p51 and Myc), initiating the inflammatory response by stimulating cell proliferation, differentiation, cell migration, and apoptosis [94]. The immune cells are also affected by this signal pathway. For instance, JAK-STAT signaling has a role in the reprogramming of the macrophage and neutrophile phenotype, namely from the proinflammatory M1 phenotype toward the anti-inflammatory M2 phenotype [95,96], and a role in neutrophile-trafficking from blood to the inflammatory site [97]. JAK-STAT also affects the differentiation of the T lymphocytes [98] and dendritic cells [99]. The JAK-STAT signaling pathway can also be inhibited by SL. Cheng and collaborators demonstrated that 6-oangeloylplenolin directly interacts with the STAT3-SH2 function domain, inhibiting the constitutive activation of STAT3 and ultimately downregulating the expression of several proinflammatory molecules, among them IL-6 [100] (Table 2). *Cynara cardunculus* leaf ethanolic extracts, mainly composed by cynaropicrin, have recently been described as having the ability to reduce 86% of IL-6 expressed in skin keratinocytes [101]. The sesquiterpene lactone antrocin, isolated from the plant *Antrodia camphorata*, inhibits the JAK2/STAT3 pathway. This is explained by the suppression of the phosphorylation of STATs and their subsequent nuclear translocations [102] (Table 2). Parthenolide, similar to its effect upon NF-kB signaling, also covalently binds to JAK2 Cys178, Cys243, Cys335, and Cys480 residues (Figure 3), all located in the SH2 domain, suppressing their kinase activity, blocking STAT protein phosphorylation, and downmodulating the expression of pro-inflammatory genes [103]. It is important to claim the preference of parthenolide to JAKs over abundant proteins, such as tubulin and actin, demonstrating a certain degree of specificity in terms of target Cys residues. The parthenolide effect upon STAT might also be explained by the role of the nucleophilic methylene-γ-lactone ring upon ROS induction, which was reported to regulate STAT signaling [104]. Other SL were also reported as having inhibitory effects in the activation of STAT proteins, such as costunolide, dehydrocostuslactone, cynaropicrin, and alantolactone through S-glutathionylation of Cys residues in the STAT3 protein (Table 2). The presence of the α-β-unsaturated carbonyl group directly interacts with GSH by Michael addition and induces a rapid drop in GSH concentration, thereby triggering S-glutathionylation of STAT3 [105]. This event impairs STAT3 phosphorylation, switching off the signaling cascade [58]. 

Table 2 identifies other sesquiterpene lactones that exerted downregulation of NF-kB, MAPK, or JAK-STAT signaling pathways, by similar molecular mechanisms, such as the ones above-mentioned. 

### 3.3. Promising Sesquiterpene Lactones under Pre-Clinical or Clinal Studies: Parthenolide, Artemisinin, and Thapsigargin 

**Figure 4:**
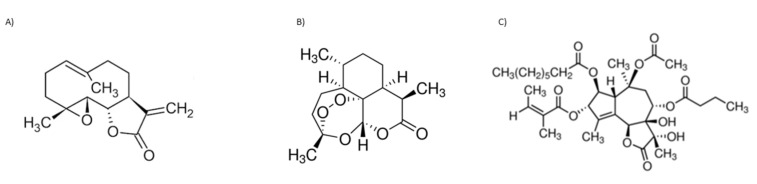
Sesquiterpene chemical structures: (**A**) parthenolide, (**B**) artemisinin, and (**C**) thapsigargin.

The known biological activity of SL prompted some SL toward pre-clinical and, eventually, clinical trials. Parthenolide (Figure 4A), artemisinin (Figure 4B), both belonging to the sesquiterpene lactone sub-group of germacranolides, and thapsigargin (Figure 4C), a guaianolide, are lead compounds in clinical trials. Although these three SL and their derivatives are used or are under clinical trials, mainly due to their anti-cancer properties, these sesquiterpene lactones will be further explored within the present review, since most mechanisms involved in tumoral responses are also crucial for anti-inflammatory activity. The inherent risk of toxicity and the lack of selectivity are of high importance and frequently impair their use. 

Parthenolide has low solubility in water and, consequently, reduced bioavailability, which hampers its potential clinical use. A Phase I trial was conducted to investigate pharmacokinetics and toxicology in a cohort of 12 adult cancer patients. Solid phase extraction and mass spectroscopy were used to evaluate parthenolide plasma concentrations. A starting dose of 1 mg/day was assessed with subsequent dose escalations to 2, 3, and 4 mg as daily oral capsules. The limit of detection in plasma was 0.5 ng/mL; no significant toxicity was detected. The low doses of this SL administered were unbale to be detected, likely due to poor bioavailability [124]. To increase solubility, several different parthenolide derivatives were constructed by the diastereoselective addition of several primary and secondary amines to the exocyclic double bond [125]. Among these semisynthetic analogues, N,N-dimethylamino-parthenolide (DMAPT) (Table 3) was selected as a leader compound according to its pharmacokinetic, pharmacodynamic, and bioavailability properties [126], and it was evaluated in a Phase I clinical trial for the treatment of hematological tumors, with a potent suppression of the STAT3 signaling pathway [127]. DMAPT fumarate salt (DMPAPT), being highly water-soluble (100x more than parthenolide), was rapidly converted to parthenolide within body fluids [127]. DMAPT was assayed in a Phase I trial against acute myeloid leukemia, acute lymphoblastic leukemia, and other blood and lymph node cancers, but clinical trials ended-up being suspended. More recently, Darwish et al. targeted parthenolide nanoencapsulation to improve drug delivery, designing polylactide co-glycolide nanoparticles conjugated with anti-CD44 and encapsulating parthenolide. The results highlighted the possibility of using nanotechnology to improve parthenolide and/or its derivatives, biological activity [128]. Over the last 5 years, there were few patents registered—regarding the elaboration of parthenolide (and derivative) nanocarriers, and their putative uses within different pharmaceutical applications (patents N. CN 110292640, 2019; N. CN108721276, 2018; N. CN1087211330, 2018; N. CN106366068, 2017; N. CN 109276553, 2017) [43].

Artemisinin (Figure 4B) is widely used worldwide to treat plasmodium strains; its biological value is recognized by the World Health Organization, with few restrictions, due to the emergence of artemisinin resistance regarding malaria treatment. As shown in recent years, artemisinin potential applications imply treatment of different infection agents. The notoriously poor solubility of artemisinin in water and oil has led to the synthesis of several chemical derivatives aimed at increasing its solubility without sacrificing the structural factors responsible for its therapeutic efficacy [129]. The different derivatives have been used in preclinical animal models and in clinical patients, such as dihydroartemisinin (DHA), artemether, artesunate, and arteether [130]. Few artesunate clinical trials focused on their anti-cancer activities have been developed [131]. Oral artesunate was safe and well tolerated up to 200 mg/day in a Phase I, dose-finding study, in 23 advanced breast cancer patients (Table 3). Stable disease, considered as a clinical benefit, was observed in 10 patients (150 and 200 mg). Progression was observed in the remaining assessable patients (n = 5) and response assessments could not be performed for eight patients [132]. Clinical and experimental studies also suggested that artemisinin and its derivatives possess potent immune-suppressive abilities to treat autoimmune and allergic diseases. A series of novel artemisinin derivatives with lower toxicity, greater water solubility, higher bioavailability, and potent immunosuppressive activity were developed, including 3-(12-*β*-artemisininoxy) phenoxyl succinic acid (SM735 [133]), 1-(12-*β*-dihydroartemisinoxy)-2-hydroxy-3-tert-butylaminopropane maleate (SM905 [134,135,136]), ethyl 2- [4-(12-*β*-artemisininoxy)] phenoxylpropionate (SM933 [137]), and 2’-aminoarteether (*β*) maleate (SM934 [138,139,140]) (Table 3)). Of note, SM934 was recently approved by the China Food and Drug Administration for a clinical trial, as a novel therapeutic agent to treat systemic lupus erythematosus (SLE). These different derivatives shared potent immunoregulatory properties, with SM934 found to preferentially promote activated T cells into early apoptosis, leaving resting T cells untouched [130], exhibiting extensive protective effects in chronic inflammation conditions, such as clinically effective corticosteroids. 

Thapsigargin structure (Figure 4C) was described in 1978 for the first time [141]. Different synthetic and semi-synthetic derivatives were developed due to their biological activities. Mipsagargin, (8-*O*-(12-aminododecanoy)-8-*O*-debutanoyl thapsigargin)-Asp-γ-Glu-γ-Glu-γGluGluOH, is a soluble licensed prodrug (G-202), https://www.drugbank.ca/drugs/DB11813, accessed on 8 December 2021, containing a cytotoxic analog of thapsigargin linked, via a carboxyl group, to the targeting peptide containing aspartic acid with potential antineoplastic activity (Table 3). Mipsagargin is converted, through cleavage of the peptide portion, into the active cytotoxic analog of thapsigargin, known to achieve higher concentration of the active agents at the tumor site while avoiding systemic toxicity [142,143]. In a recent clinical study with 25 patients, mipsagargin was well tolerated and promoted prolonged disease stabilization in patients with hepatocellular carcinoma that had progressed in prior treatment with sorafenib [144]. Thapsigargin dehydrocostuslactone is another analogue synthesized by formation of a five-membered ring though a 1,8-addition with a Grignard reagent, with completion of a regio- and stereoselective-butyrolactone via an epoxide opening with dilithioacetate, and introduction of three exocyclic methylene groups. This analogue inhibited TNF-α-induced NF-κB activation, and inhibited the JAK/STAT3 pathway in breast cancer cells [133,145]. 

The presented clinical trials inspire new strategies for sesquiterpene lactone therapeutic valorization. The findings seem promising; however, questions remain about their efficacy as anti-inflammatory drugs. The concentrations upon which SL are pharmacologically active are not well-described, reinforcing the importance of studying the pharmacokinetic (ADME—absorption, distribution, metabolism, excretion) profiles of these molecules.

## 4. Enhancement of the Biological Activity of Sesquiterpene Lactones

Anti-inflammatory activities, as well as modulations of the immune systems, make SL attractive in pharmaceutical use. The possibility in biosynthetic engineering, as well as the impact of new extraction methodologies to achieve SL-enriched extracts, are now being addressed, in the perspective of two valuable tools for medicinal use of sesquiterpene lactones.

### 4.1. Metabolic Engineering for Sesquiterpene Lactones Specialized Production

Over the last few years, researchers have taken advantage of the advances in genomics, transcriptomics, and metabolomics, which have resulted in a greater understanding of the pathways and regulatory mechanisms involved in the biosynthesis of specialized terpenoids. The presence of gene clusters has impacted technology in unravelling biosynthetic pathways, not yet clarified, favoring the metabolic engineering of target compounds. Regarding the gene clusters metabolized so far, we can mention those related to the biosynthesis of terpenoids containing enzymes that converge to several terpenoid structures that can be modified by additional enzymes, opening a range of possibilities for other compounds, such as SL. For high throughput production of SL, increasing total production of their main precursors, isopentenyl diphosphate (IPP) and dimethylallyl diphosphate (DMAPP) are vital prerequisites. Furthermore, elucidating new ways to replenish IPP and DMAPP are different, yet effective methods to improve sesquiterpene lactone production. Modification at the level of the MEP/MVA pathways is of great importance to obtain improved content of the precursors; modifications in these pathways are usually via the regulation of specific enzymes. Negative regulatory genes could be silenced from the biosynthetic line, and some gene modules could undergo recombination or replacement by auxiliary genes [146]. The translation of modular genetic pathways with an ample supply of precursors has been successfully applied to producing artemisinin. Another complementary approach involves heterologous production platforms. Malhotra et al. used double tobacco transgenic lines with a three-fold enhancement of IPP, resulting in a higher expression and an efficient photooxidation of dihydroartemisinic acid to artemisinin, with great biological activity [147]. Equivalent approaches are easily upscaled to the industry’s productivity needs, making compounds of interest available, with competitive production costs.

### 4.2. Non-Conventional Sesquiterpene Lactone Extraction and Purification Processes

Solid–liquid extraction is a fundamental key in the assessment of the biological potential of SL. Throughout the years, several extraction methodologies and extraction solvents have been applied to the extraction of SL, with a focus on improving their biological potential. Recently, to increase the anti-inflammatory activity, non-conventional and green extraction methodologies, such as supercritical CO_2_, were applied upon extraction of SL [148]. Besides the providing efficiency, when compared to the traditional one, supercritical CO_2_ presented a higher selectivity (53 µg/mg) and allowed the reduction of organic solvents consumed [148]. The produced extracts were tested for their anti-inflammatory potential; a decrease on the activity of the calcineurin/Crz1, the *S. cerevisiae* orthologue of the human nuclear factor of the activated T cells (NFAT) pathway was observed. The SFE extract, rich in 11β,13-dihydrolactucin, lactucin, 11β,13-dihydrolactucopicrin, and lactucopicrin, was able to inhibit 61.74 ± 6.87% of the transcription factor activity with a concentration of 50 µg/mL, an inhibition rate in the same range of those observed for the immunosuppressant pharmaceutical drug FK506, also known as tacrolimus [148], indicating the possibility of application of this extraction method on the production of sesquiterpene lactone-enriched anti-inflammatory extracts using green technologies at reduced costs. Moreover, a SFE purified fraction, containing a mixture of 8-deoxylactucin and 11β,13-dihydro-8-deoxylactucin, presented enhanced anti-inflammatory activity, based on the capacity to decrease the activity of the calcineurin/NFAT orthologue, IC_50_ of 7.2 ± 3.15 µg/mL [148].

Nevertheless, an improvement on biological potential can also be achieved by separation processes, as the classic and solid phase extraction and/or liquid extraction. Liquid extraction, although representing extra organic solvent consumption, is still one of the main procedures for compound purification, where a higher biological activity of active fraction can be achieved. Ferrari et al. studied the anti-inflammatory activity of ethanol extract from aerial parts of *Lychnophora trichocarpha* and its ethyl acetate fraction [149]. Topical treatment with ointments containing ethanol extract, its ethyl acetate fraction and SL, lychnopholide (Lyc), and eremantholide C reduced carrageenan-induced mice paw edema. Comparative to the ethanolic extract and ethyl acetate fraction, the latter promoted low paw swelling (0.062 ± 0.04 mm) when compared to the initial extract (0.112 ± 0.05 mm), with these results correlating to the content of SL [149]. Recently, Bras et al. studied the application of organic solvent nanofiltration on the purification of cynaropicrin from *Cynara cardunculus* leaf extracts, where the removal of low molecular weight compounds resulted in an increase of extract bioactivity, expressed in terms of fibroblast Bj5-ta cell viability [150].

## 5. Conclusions

The chemical structures of SL allow for different target interactions. The α-methylene-γ-lactone moiety is the structural feature with higher relevance in regard to the bioactivity of SL. SL are able to modulate different cellular responses, with a recognized impact on immune modulation and inflammatory response, a production decrease of IL-6, IL-8, and the TNF-α of neutrophils, inhibition of macrophage LPS-induced nitric oxide production, eosinophils reduction, favoring the degranulation of basophils, inhibiting T lymphocyte receptors, and suppressing lymphocyte proliferation, as cell reducing dendritic cell maturation. At the molecular level, SL impact three mainly inflammatory signal pathways, NF-kB, MAPK, and JAT-STAT, of non-immune cells, such as epidermal keratinocytes. The gene expression resulting by the action of these signal pathways will affect the proliferation, survival, maturation, differentiation, chemoattraction, and trafficking of immune cells.

Appropriate in vivo studies addressing the anti-inflammatory potential of sesquiterpene lactones, as well as evaluating their toxic properties, genotoxicity, and embryotoxicity, are needed. The slightly different chemical structures of SL might induce different biological activities; for this reason, it is important to properly understand the toxicological profiles of these compounds. The efficacy of biological activity is missing link between toxicity and selectivity, which needs to be potentiated. Comprehensive clinical trials are needed to establish the efficacy of SL and/or their derivatives, as safe anti-inflammatory drugs.

In the near future, there needs to be more advances in pharmacokinetics and nanotechnology. Although with some recent developments, the solubility enhancement will dictate the extension of sesquiterpene lactone use, and the eventual routes of administration. All evaluations will lead to new knowledge and strategies regarding the biological and clinical relevance of sesquiterpene lactones, in regard to inflammation response, or their related comorbidities.

## Figures and Tables

**Table 1:** SL described as having regulatory functions upon the immune system, highlighting immunoregulatory actions within acquired and innate responses.

Sesquiterpene Lactone	Action	Reference
Acquired Immune Response		
Reduction of T cells production		
Arglabin Grosheimin Agracin Parthenolide Estafiatin	↓ TCR	[45]
Artemether (an artemisinin derivative)	↓ IL-2, interferon-γ (IFN- γ), TCR ↓ phosphorylation of ERK1/2, JNK, and p38	[44]
7-hydroxyfrullanoide	↓ IL-2, ↑↑ Ca^2+^ ⇒ ↓ CD4^+^ ↓ IL-6, IFN- γ	[46]
Cynaropicrin	↓ proliferation of CD4^+^ and CD8^+^ T- and B- lymphocytes	[34]
Deoxyelephantopin Isodeoxyelephantopin	↓ lymphocytes	[57]
**Innate Immune Response**		
**Macrophage Inhibition**		
Tagitinin C, F and A	↑ neutrophils apoptosis, ↓ IL-6, ↓ IL-8, ↓ TNF-α	[47]
**Neutrophils Inhibition**		
Diacethylpiptocarphol Hirsutinolides	↓ neutrophil infiltration	[53]
Lychnopholide Eremantholide C Goyazensolide	↓ neutrophil infiltration, ↓ TNF-α	[54]
Budlein A	↓ Neutrophil recruitment, ↓ Il-1β and TNF-α mRNA	[55]
Alantolactone	↓ TNF-α, ↓ IL-6 and ↓IL-17A,	[56]
Costunolide	↓ Neutrophil recruitment,	[55]
**Eosinophils Reduction**		
Alantolactone Costunolide Dehydrocostuslactone	↓ Th2 cytokines (IL-4 and IL-3)	[50]
Damsin Neoambrosin	Eosinophils	[58] [59]

**Table 2:** SL described as having roles in inflammatory signaling mechanisms, namely NF-kB, MAPK, and JAK-STAT.

Inflammatory Signaling Mechanism	SL	Downstream Effect	References
**NF-kB**	Parthenolide Heliangin Vlasouliolides E-I Damcin Ambrosin Coronopilin 7-hydroxy frullanolide Budlein A Secoeudesma sesquiterpenes lactone A Costunolide Gaillardin Micheliolide	↓ RelA phosphorylation, ↓ NF-kB DNA binding ↑ IkBα, ↓ NF-kB translocation; ↓ IL-8	[89,106] [107] [108] [109] [110] [111] [112] [55] [113] [114,115] [116]
	Artemisinin Cnicin	↑ IkBα, ↓ NF-kB translocation, ↓ iNOS	[78] [117]
	Santamarin	↓ NF-kB, ↑ HO-1, ↓ NO, PGE2, TNF-α, IL-1β	[82]
	Dehydrocostus lactone	↓ NF-kB, ↓ IFR3, ↓ COX-2, ↓ IIP-10	[83]
	Lactucopicrin	↓ importin-α3 ↓ NF-kB	[118]
**NF-kB and MAPK**	Alantolactone Torilin	↓IKK, ↑ IkBα, ↓ NF-kB, ↓ AP-1 (MAPK), ↓ iNOS, ↓ COX-2	[91] [119]
	Ginkgolides	↓ PAF-induced platelet aggregation	[81]
**MAPK**	Parthenolide	↓ ERK1/2 phosphorylation	[89]
	Achillolide A	↓ SAPK/JNK and p44/p42 MAPK phosphorylation	[90]
	2α-hydroxyl-3β-angeloylcinnamolide	↓ ERK1/2, ↓p38 and ↓JNKs phosphorylation	[120]
**MAPK and JAK/STAT**	Damsin Neoambrosin	↓ ERK1/2, ↓ STAT3, ↓ TNF-α, ↓ IL-6 and ↓ IL-12	[121]
**JAK/STAT**	Parthenolide Antrocin Costunolide Dehydrocostuslactone Cynaropicrin Alantolactone Damcin	↓ STAT phosphorylation (S-glutathionylation of Cys residues)	[103] [102] [105] [122] [109] [123]
	6-Oangeloylplenolin	↓ STAT3 activation (block STAT3-SH2 function domain), ↓ IL-6	[100]

**Table 3:** Sesquiterpene lactones and their derivatives with clinical relevance.

Sesquiterpene Lactone or Derivative	Clinical Study	References/ ClinicalTrals.gov Identifier
Dimethyl-amino-parthenolide (LC-1) 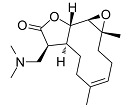 CAS number: 870677-05-7	Phase I clinical trials	[124]
Artesunate (DB09274) 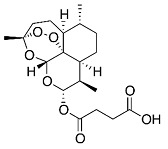 CAS number: 80155-81-3	Advanced breast cancer High grade vulvar intraepithelial neoplasia Phase I clinical trials Phase II/III Colorectal cancer	NCT00764036 [132] NCT03792516 NCT04098744 NCT03093129
2’-aminoarteether (*β*) maleate (SM934) 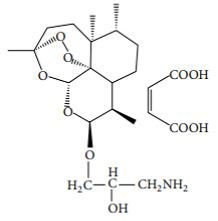 CAS number: 133162-25-1	Licensed drug Systemic lupus erythematosus	[138,139,140] Approved by the China Food and Drug Administration
Mipsagargin (G-202) 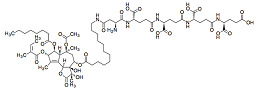 CAS number: 1245732-48-2	Licensed drug Advanced solid tumors	NCT01056029 [143] https://www.drugbank.ca/drugs/DB11813 (last accessed on 7 January 2022
